# Design and Fabrication of Micro/Nano Sensors and Actuators, Volume II

**DOI:** 10.3390/mi15060667

**Published:** 2024-05-21

**Authors:** Weidong Wang, Yong Ruan, Zaifa Zhou, Min Liu

**Affiliations:** 1 School of Mechano-Electronic Engineering, Xidian University, Xi’an 710071, China; mliu_12@stu.xidian.edu.cn; 2 Department of Precision Instruments, Tsinghua University, Beijing 100084, China; ruanyong@mail.tsinghua.edu.cn; 3 School of Electronic Science and Technology, Southeast University, Nanjing 210096, China; zfzhou@seu.edu.cn

Microelectromechanical system (MEMS) sensors are a miniaturized sensor technology that integrates sensors with microelectronic components using microelectromechanical system manufacturing technology [1,2]. MEMS sensors typically consist of micromechanical structures, sensing circuits, and signal processing circuits, which can be used to measure and detect various physical quantities, such as pressure [3,4], temperature [5,6], acceleration [7,8], angular velocity [9,10], and humidity [11,12]. These micromechanical structures are typically composed of microsprings [13], thin films [14], and cantilever beams [15], and their dimensions typically range from micrometers to millimeters. When external physical quantities act on these micromechanical structures, they will undergo deformation or vibration, thereby changing parameters such as current, voltage, or capacitance in the circuit and generating corresponding electrical signals [16,17,18]. The miniaturization and integration of MEMS sensors have made them applicable in various fields, such as the automotive industry [19], healthcare [20], and aerospace [21], and have provided important support for the development of intelligent systems [22] and the Internet of Things [23].

This Special Issue contains 10 papers, mainly introducing various MEMS sensors, including inertial devices such as accelerometers and gyroscopes (Contributions 1 and 2), differential pressure sensors (Contribution 3), optical analysis of microfluidic technology (Contribution 4), thermoelectric temperature sensors (Contribution 5), thin film thermal flow sensors (Contribution 6), thermocouple sensors (Contribution 7), resonators (Contribution 8), microwave sensors (Contribution 9), and skin friction sensors (Contribution 10).

In particular, Zhang et al. (Contribution 1) presented a MEMS electrochemical angular accelerometer with a silicon-based four-electrode structure. Numerical simulations were conducted to optimize the geometrical parameters of the silicon-based four-electrode structure. These simulations demonstrated that increases in fluid resistance and cathode area can expand the working bandwidth and improve the device sensitivity, respectively. Zhang et al. (Contribution 2) proposed a weak capacitance detection circuit for a micro-hemispherical gyroscope based on common-mode feedback fusion modulation and demodulation. The input common-mode feedback is applied to the circuit to address the input common-mode voltage drift caused by both parasitic capacitance and gain capacitance. Secondly, a low-noise, high-gain amplifier is used to reduce the equivalent input noise. Thirdly, the modulator–demodulator and filter are introduced to the proposed circuit to effectively mitigate the side effects of noise. Yasunaga et al. (Contribution 3) designed a MEMS differential pressure sensor with a dynamic pressure canceler. They proposed a sensor “cap” that cancels the wind effect and noise by utilizing the airflow around a sphere. In addition, a height estimation method was developed based on a discrete transfer function model. This sensor can be used for outdoor drones and wave height measurement. Qin et al. (Contribution 4) analyzed the optical path in non-uniform refractive index media in surface acoustic wave (SAW) microfluidic chips and proposed a MEMS SAW device based on a solid medium. The device can control the focal length by changing the voltage and adjusting the sharpness of the micrograph. Wang et al. (Contribution 5) proposed a flexible temperature sensor based on graphene fibers. Graphene fibers (GFs) are synthesized by a facile microfluidic spinning technique using a green reducing agent (vitamin C). The sensor has good scalability in length, and its sensitivity can increase with the number of p–n thermocouples. Chen et al. (Contribution 6) established a finite element analysis model of thin-film heat flow meters based on finite element simulation methods, and a double-type thin-film heat flow sensor based on a copper/concentrate thermopile was made. Moreover, the device exhibited a dual-use characteristic, which considerably broadened the scope of its applications and service life and introduced a novel approach to measuring heat flow density in the intricate environment on the surface of the aeroengine. Han et al. (Contribution 7) studied the impact of different annealing times on the cyclic characteristics of ceramic oxide thin film thermocouples. The results show that when the annealing temperature is held constant, extending the annealing time can improve the properties of the film, increase its density, slow down oxidation, and enhance the thermal stability of the thermocouple. Liu et al. (Contribution 8) conducted in-depth analysis on the design and optimization of hemispherical resonators. The geometric parameters that significantly contribute to the performance of the resonator were determined via a thermoelastic model and process characteristics. And then they proposed an optimization method for the MEMS polysilicon hemispherical resonator based on PSO-BP and NSGA-II. Kim et al. (Contribution 9) designed a multi-metal crack detection (MCD) microwave sensor with two independent complementary split-ring resonators (CSRRs) loaded onto the substrate-integrated waveguide (SIW). The concentrated electromagnetic field of the SIW improves the Q-factor of the CSRRs. The proposed multi-MCD sensor can detect cracks of different widths and depths with a high Q-factor and high precision. Guo et al. (Contribution 10) studied the three-dimensional numerical simulation and structural optimization for a MEMS skin friction sensor in hypersonic flow. The objective of this study is to investigate the influence of sensor-sensitive structure on wall-flow characteristics and friction measurement accuracy. To this end, the two-dimensional and three-dimensional numerical models of the sensor in the hypersonic flow field based on computational fluid dynamics (CFD) are presented. Finally, the sensor-sensitive unit structure’s design criterion is obtained to improve skin friction’s measurement accuracy.

We would like to take this opportunity to thank all the authors who have submitted their papers to this Special Issue and to thank all the reviewers for their contributions in evaluating the submitted papers and for their efforts in improving the quality of the manuscripts.

## References

[1] de Groot W.A., Webster J.R., Felnhofer D., Gusev E.P. (2009). Review of device and reliability physics of dielectrics in electrostatically driven MEMS devices. IEEE Trans. Device Mater. Reliab..

[2] Zhu J., Liu X., Shi Q., He T., Sun Z., Guo X., Lee C. (2019). Development trends and perspectives of future sensors and MEMS/NEMS. Micromachines.

[3] Liu Y., Jiang X., Yang H., Qin H., Wang W. (2023). Structural Engineering in Piezoresistive Micropressure Sensors: A Focused Review. Micromachines.

[4] Nallathambi A., Shanmuganantham T., Sindhanaiselvi D. (2018). Design and analysis of MEMS based piezoresistive pressure sensor for sensitivity enhancement. Mater. Today Proc..

[5] Kose T., Azgin K., Akin T. (2016). Design and fabrication of a high performance resonant MEMS temperature sensor. J. Micromech. Microeng..

[6] Algamili A.S., Khir M.H., Ahmed A.Y., Rabih A.A., Ba-Hashwan S.S., Alabsi S.S., Al-Mahdi O.L., Isyaku U.B., Ahmed M.G., Junaid M. (2022). Fabrication and Characterization of the Micro-Heater and Temperature Sensor for PolyMUMPs-Based MEMS Gas Sensor. Micromachines.

[7] Liu M., Wu X., Niu Y., Yang H., Zhu Y., Wang W. (2022). Research Progress of MEMS Inertial Switches. Micromachines.

[8] Singh V., Kumar V., Saini A., Khosla P.K., Mishra S. (2020). Design and development of the MEMS-based high-g acceleration threshold switch. J. Microelectromech. Syst..

[9] Ren J., Zhou T., Zhou Y., Li Y., Su Y. (2023). An In-Run Automatic Mode-Matching Method with Amplitude Correction and Phase Compensation for MEMS Disk Resonator Gyroscope. IEEE Trans. Instrum. Meas..

[10] Li Z., Cui Y., Gu Y., Wang G., Yang J., Chen K., Cao H. (2023). Temperature drift compensation for four-mass vibration MEMS gyroscope based on EMD and hybrid filtering fusion method. Micromachines.

[11] Dennis J.O., Ahmed A.Y., Khir M.H. (2015). Fabrication and characterization of a CMOS-MEMS humidity sensor. Sensors.

[12] Hassan A.S., Juliet V., Raj C.J.A. (2018). MEMS bas ed humidity sensor with integration of temperature sensor. Mater. Today Proc..

[13] Zhang F., Wang C., Yuan M., Tang B., Xiong Z. (2017). Conception, fabrication and characterization of a silicon based MEMS inertial switch with a threshold value of 5 g. J. Micromech. Microeng..

[14] Song X., Liu H., Fang Y., Zhao C., Qu Z., Wang Q., Tu L.C. (2020). An integrated gold-film temperature sensor for in situ temperature measurement of a high-precision MEMS accelerometer. Sensors.

[15] Xu Q., Rocha R.T., Algoos Y., Feron E., Younis M.I. (2024). Design, simulation, and testing of a tunable MEMS multi-threshold inertial switch. Microsyst. Nanoeng..

[16] Zhang J., Chen J., Li M., Ge Y., Wang T., Shan P., Mao X. (2018). Design, fabrication, and implementation of an array-type MEMS piezoresistive intelligent pressure sensor system. Micromachines.

[17] Zhao Y., Wang Z., Tan S., Liu Y., Chen S., Li Y., Hao Q. (2022). Dependance of Gauge Factor on Micro-Morphology of Sensitive Grids in Resistive Strain Gauges. Micromachines.

[18] Cao Y., Xi Z., Yu P., Wang J., Nie W. (2015). A MEMS inertial switch with a single circular mass for universal sensitivity. J. Micromech. Microeng..

[19] Zhang W., Hao C., Zhang Z., Yang S., Peng J., Wu B., Wang R. (2020). Vector high-resolution marine turbulence sensor based on a MEMS bionic cilium-shaped structure. IEEE Sens. J..

[20] Baik J., Seo S., Lee S., Yang S., Park S.M. (2021). Circular radio-frequency electrode with MEMS temperature sensors for laparoscopic renal sympathetic denervation. IEEE Trans. Biomed. Eng..

[21] Ghazali M.H.M., Rahiman W. (2023). Fuzzy-Oriented Anomaly Inspection in Unmanned Aerial Vehicle (UAV) Based on MEMS Accelerometers in Multi-Mode Environment. IEEE Trans. Instrum. Meas..

[22] Podder I., Fischl T., Bub U. (2023). Artificial Intelligence Applications for MEMS-Based Sensors and Manufacturing Process Optimization. Telecom.

[23] Xu Q., Yang Z., Sun Y., Lai L., Jin Z., Ding G., Wang J. (2017). Shock-resistibility of mems-base d inertial microswitch under reverse directional ultra-high g acceleration for IoT applications. Sci. Rep..

